# Lung Cancer Presenting as Abdominal Skin Metastasis: An Unsolved Case With Extensive Literature Review

**DOI:** 10.7759/cureus.91014

**Published:** 2025-08-26

**Authors:** Jyoti Verma, Josephain K, Jyotsna N Bharti, Hemant Kumar Singh, Mithilesh Arumulla

**Affiliations:** 1 Department of Pathology and Laboratory Medicine, All India Institute of Medical Sciences, Mangalagiri, Mangalagiri, IND; 2 Department of Surgical Oncology, All India Institute of Medical Sciences, Mangalagiri, Mangalagiri, IND; 3 Department of Radiology, All India Institute of Medical Sciences, Mangalagiri, Mangalagiri, IND

**Keywords:** adenocarcinoma, lung cancer, metastasis, presentation, skin

## Abstract

Skin metastasis is the spread of malignant cells to the skin from a primary malignancy. It is quite uncommon for skin or cutaneous metastasis of internal malignancies to manifest as an initial presentation. It occurs in about 5.3% of patients with internal malignancies, representing 2% of all skin tumors. Lung cancer (LC) has the highest incidence and is the most common cause of cancer death worldwide. Metastasis to the skin should be carefully investigated to rule out a metastatic manifestation of an occult primary site tumor. It mostly occurs late in the course of the disease and indicates a poor prognosis. We report a case of a 65-year-old male presenting with multiple abdominal skin nodules as an initial uncommon presentation of unknown primary, along with a review of the literature for which we performed a Medline search for articles on cutaneous metastases from internal malignancies, including LC, using PubMed, and a manual search of pertinent references and textbooks.

## Introduction

Skin metastasis as a primary manifestation of internal malignancy is rarely seen [1]. In 2022, lung cancer stood as the most frequently diagnosed cancer and the primary cause of cancer-related deaths on a global scale, with approximately 2.48 million new cases and 1.8 million deaths, respectively [2]. Lung adenocarcinoma (LAC) commonly metastasizes to the hilar lymph nodes, liver, adrenal glands, brain, and bone, but skin metastasis is infrequent and may present as an early sign of underlying malignancy or a clue to tumor recurrence [3]. It is usually associated with advanced lung cancer (LC) and poor prognosis. Hence, there should be a high index of clinical suspicion in cases with cutaneous presentation. The final diagnosis is established by a thorough clinical workup and histopathology examination (HPE) to identify the occult primary site. In most cutaneous deposits, survival time is usually less than a year, while the reported mean survival for skin metastases of LAC is 2.9 months [4].

## Case presentation

**Figure 1:**
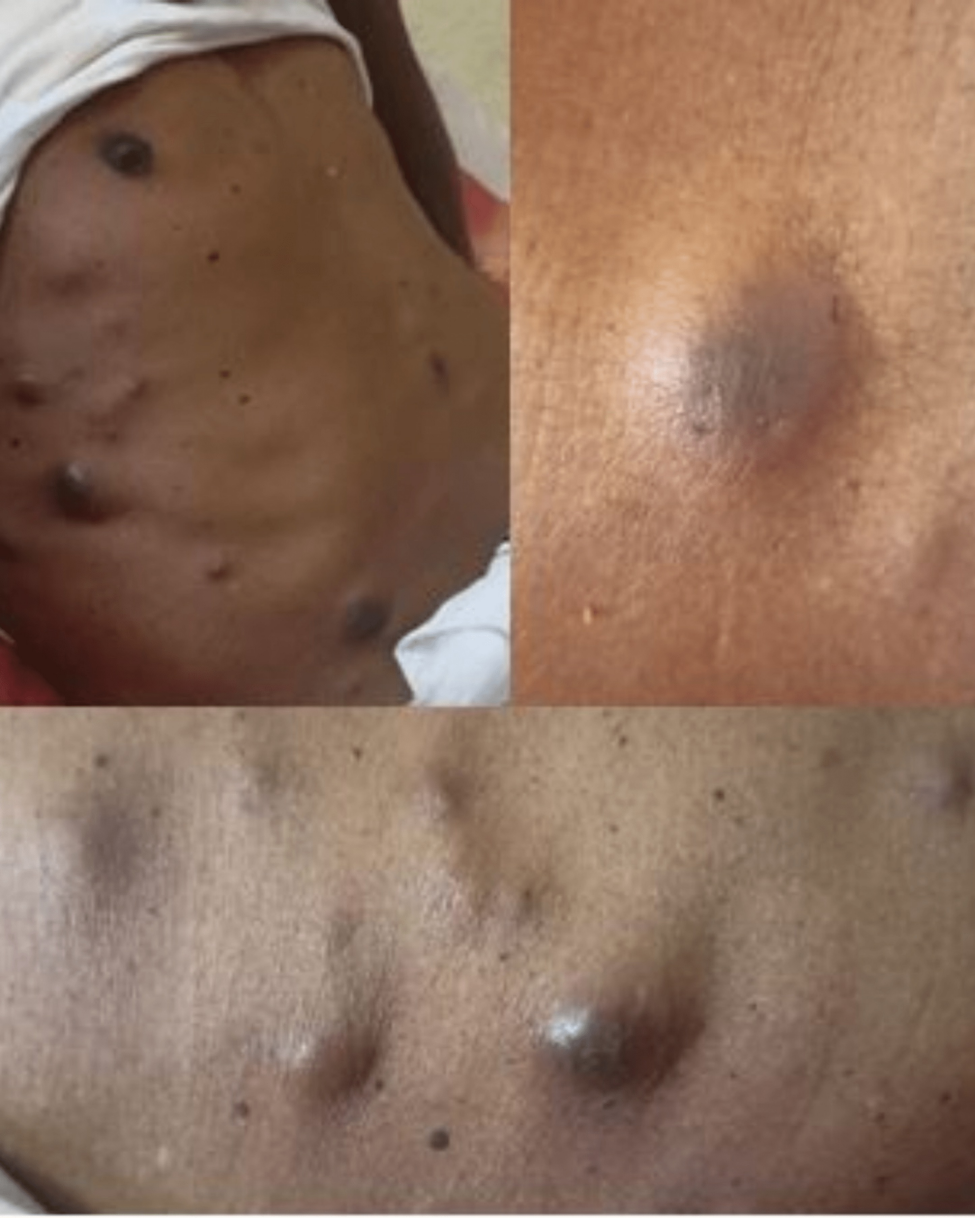
Multiple, firm, non-tender cutaneous nodules over the abdomen varying in size from 0.5 cm to 2 cm.

A 65-year-old male presented to the oncosurgery department with multiple abdominal skin nodules for six months and a mild backache (Figure 1).

**Figure 2:**
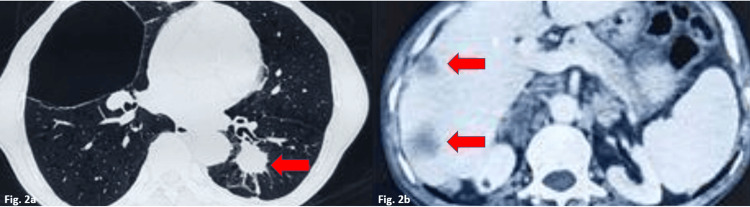
2a-Contrast tomography (CT) images of the chest showing ill-defined heterogeneous enhancing hypo-dense lesions with speculated margins in the left lower lobe measuring 26x18 mm, likely of primary malignant etiology. 2b - CT abdomen showing multiple hypodense heterogeneously enhancing lesions in both lobes of the liver and bilateral, bulky, moderately enhancing adrenal glands, most likely metastasis.

He had mild breathlessness and a smoking history of 10 years. He had quit smoking two years back. On examination, these nodules were multiple, firm, and non-tender, varying in size from 0.5 cm to 2 cm. Hematological parameters were within normal limits. High-resolution computed tomographic scanning (HRCT) of the chest showed an ill-defined, heterogeneously enhancing, hypo-dense lesion with speculated margins in the left lower lobe measuring 26x18 mm, likely malignant, along with diffuse emphysematous changes in various segments (Figure 2a).

A few enlarged pre-tracheal, subcarinal, and hilar lymph nodes were noted. A CT scan of the abdomen showed multiple hypodense heterogeneously enhancing lesions in both lobes of the liver and bilateral adrenal glands and a few enlarged retroperitoneal lymph nodes (Figure 2b).

**Figure 3:**
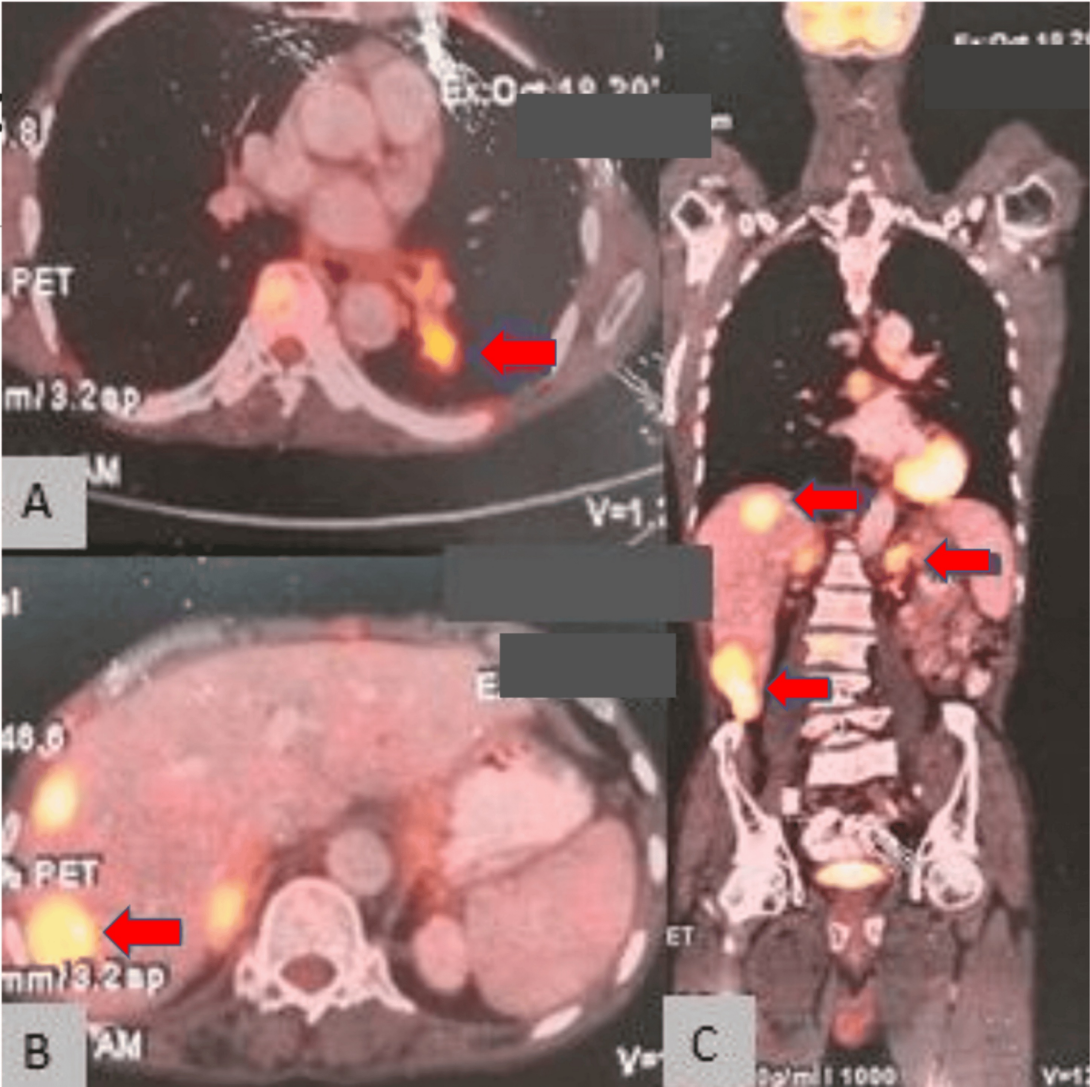
On Fluoro-deoxyglucose-positron emission tomography (FDG-PET), a cross-sectional view depicting intense uptake in a nodular mass lesion in the left lower lung lobe (A). Cross-sectional view depicting intense uptake in the liver nodules (B), a longitudinal section depicting intense uptake in the brain lesions, and a few retroperitoneal lymph nodes (C).

Fluoro-deoxyglucose (FDG) positron emission tomography (PET) findings confirmed CT findings showing intense uptake in nodular lesions in the left lower lobe of the lung, in bilateral adrenals, liver nodules, and brain lesions (Figure 3).

**Figure 4:**
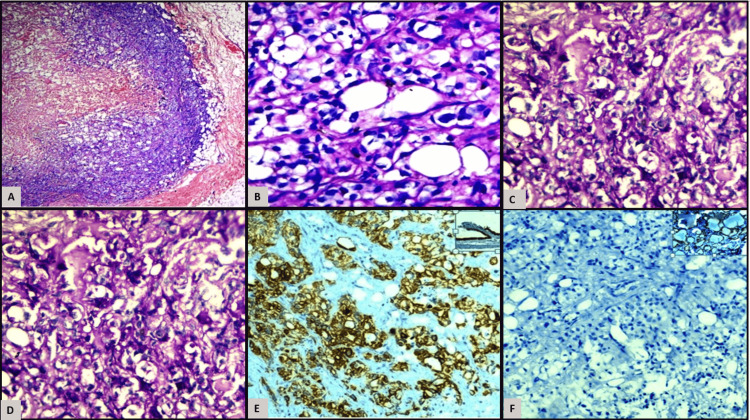
(A) Biopsy from skin nodule showing peripherally preserved tumor cells and central hemorrhagic necrosis (x2000, H&E); (B)-tumor cells with clear abundant cytoplasm and central nuclei (x400, H&E); (C)-tumor cells showing periodic acid Schiff (PAS) positivity in the tumor cells; (D)-tumor cells showing focal faint diastase PAS-positive cells depicting mucinous content in the cells; (E)-tumor cells depicting cytokeratin 7 (CK) cytoplasmic and membranous positivity in the tumor cells with an inset displaying CK positivity in the epithelium as a control; (F)-tumor cells depicting thyroid transcription factor (TTF1) negativity in tumor cells with an inset displaying TTF1 nuclear positivity in thyroid tissue as a control.

An excision biopsy of the skin nodule was sent to the pathology department for histopathological examination (HPE) (Figures 4a-4f).

The specimen showed grey-white to grey-brown skin-covered tissue measuring 0.8 x 0.5 x 0.4 cm. The HPE showed a clearly defined tumor in the layer of skin beneath the surface, with a center made up of fine, interconnected blood vessels, bleeding, and dead tissue. The peripheral viable zone showed atypical clear cells arranged in nests, lobules, and sheets.

These cells exhibited moderate nuclear pleomorphism, with small round nuclei that are either centrally or eccentrically located, dispersed chromatin, and conspicuous nucleoli. Their cytoplasm was eosinophilic, clear to vacuolated, and had distinct cell borders. Tumor cells were periodic acid-Schiff-diastase (PAS) and diastase PAS (D-PAS) positive, highlighting mucin.

The skin nodule biopsy was reported as metastatic clear cell malignancy with mucinous content. Immunohistochemistry (IHC) was applied to exclude/confirm primaries from the lung, renal, and gastrointestinal tissues. Tumor cells were positive for CK-7 but negative for CK 20, TTF1, synaptophysin, HMB45, CD10, Napsin A, CDX2, CD10, and S100, thus excluding various other primaries, including gastrointestinal adenocarcinoma, renal cell carcinoma, lung squamous cell carcinoma, and PEComa.

After the IHC application, the case was reported as metastatic adenocarcinoma of the lung. The patient did not undergo a lung mass biopsy, and when inquired about, the patient died three months after the diagnosis was obtained due to his worsening condition. Hence, this case remained unsolved, as no definite biopsy from the lung could be performed.

## Discussion

LC is the leading cause of morbidity and mortality, with the highest incidence of cancer-related deaths accounting for about 25% and LACs at around 40% [5,6]. In a population-based study done in 2022, LCs were found to be the most common malignancy, with a reported incidence of cutaneous metastasis of around 1 in 10 patients; however, there are various primary solid malignancies leading to cutaneous involvement, the most common being breast, followed by lung. Other common sites include the liver, adrenal glands, bone, kidney, and brain. It presents mostly as painless cutaneous nodules, leading to delayed diagnosis. [7] Cutaneous lesions are frequent in later advanced stages of malignancy but are seldom observed as primary metastatic manifestations.

Abdelazeem B et al. [8] in 2021 tried to perform a National Library of Medicine database search of journal articles on cutaneous metastases from internal malignancies, including LC, utilizing PubMed and searching for appropriate references and textbooks, and came across six retrospective analyses, two review articles, 20 case reports, and two database searches, which included a SEER Research Database, including nine Registries (1975-2018). A Medline PubMed search by Mollet et al. (2009) included 45 articles, which comprised 22 retrospective case studies, 16 case reports, one prospective study, and six review articles [9]. Total case reports and case series from 1986 until the present are listed in Table 1 [6,10-26].

**Table 1:** Enlisted case reports and series on cutaneous metastasis of lung carcinomas. EMA: Epithelial membrane antigen, CEA: Carcinoembryonic antigen, GCDFP: Gross cystic disease fluid protein, PCK: Pan-cytokeratin, NAD: Nothing abnormal detected, SCC: Squamous cell carcinoma, PDSCC: Poorly differentiated squamous cell carcinoma, MDSCC: Moderately differentiated Squamous cell carcinoma, IHC: Immunohistochemistry

Author	Age	Sex	Smoking History	Symptoms & Duration	Radiology	Lung Cancer Type	IHC Results
Falbo F.et al. [10]	82	M	Smoker, HT	Round elevated nodule, ~ 50 mm in diameter, surrounded by erythematous brownish skin, prone to bleeding from a central ulcerated area for four months.	CT-infiltrating subcarinal neoplasm measuring 42 × 29 × 50 mm distorting the middle bronchus and dislocating the esophagus to the left.	Adenocarcinoma (ACC)	(CK7+, CK20−, TTF1−, Napsin A−, CDX-2−, Ki67 60%).
Wang J. et al. [11]	52	F	Exposed to passive smoking due to her husband	Raised skin mass with swelling and erythema for three weeks.	MRI: Soft tissue mass upper back behind chest of 10 cm size.	ACC	CK, TTF1, Pan CK, +ve, P63, CK20, ER, HER2neu, p16, GATA3 -ve
Zhong et al. [12]	62	M	Nonsmoker	Rapidly growing skin lesion on left back dome -shaped, purplish-red, and firm soft tissue mass with ulceration and hemorrhagic crust, 4 cm × 4 cm on the left back for two months duration.	CT: Left upper lobe lung cancer with mediastinal and hilar lymph nodes.	ACC	CK7, TTF1, Napsin A, Vimentin +ve CK20, CD5/6-ve Ki67 90%
Tvedten E. et al. [6]	75	F	Chronic smoker but had quitted	The patient presents with tender soft tissue masses in the right mid-back, peri-umbilical area, and right chest wall/superior breast.	CT chest: a spiculated nodule in the right upper lobe, scattered solid and ground-glass nodules, a lytic expansile lesion in the T10 vertebral body, as well as soft tissue masses throughout both breasts and the anterior abdominal wall. CT abdomen: cystic lesion at costophrenic angle, hypodense lesion in liver, necrotic lesion in spleen.	ACC	CK7 +ve, CK20, TTF1, Napsin, PAX8, CDX2 -ve
Sharma ji et al. [13]	66	F	Smoker	Breathlessness for two years and multiple skin nodules, firm, non-tender skin colored 1-4 cm over the chest, back, axilla, head, and neck with bilateral neck nodes for three months.	CT image of the thorax showing a mass (marked with a white arrow) in the peri-hilar superior segment of the right lower lobe. On CECT head, moderately heterogeneously enhancing lesion causing destruction of adjacent bones. A bone scan was done, suggesting multiple osteoblastic lesions in the D6 vertebra, pelvis, left humerus, right temporoparietal region, and rib.	ACC	CK7, TTF1 +ve, CK20-ve
Hashimoto K. et al. [14]	93	M	NA	Rapid increase in pain and size of shoulder mass with restricted mobility measuring 10 cm × 8 cm with overlying red and tender skin.	MRI showed a low intensity on T1-weighted images and high intensity on T2-weighted images.	ACC	CAM5.2, CK7, and TTF +ve
Wang Y. et al. [15]	49	F	Nonsmoker	Multiple painful zosteriform vesicle-like papules 0.5-1.0 cm localized on the left breast.	NA	ACC	CK, CK7, TTF1, EMA +ve CK20, CEA, GCDFP -ve Ki 67 45-50%
Mc Sweeney WT. et al., [16]	69	F	lifelong smoker	rapidly growing lesion on her central chest for two months and had increased in size.	CT chest revealed lung malignancy, with a spiculated right apical mass measuring 48 × 30 × 29 mm, extending to the mediastinal pleura and in proximity to the esophagus. A CT brain revealed six cerebral metastases.	Non-Small Cell Lung Cancer	GATA3 and CK AE1/AE3 positive p63, CK5/6, TTF-1, Napsin negative On IHC, typification was unsuccessful.
Khaja M. et al. [17]	(1) 80 (2) 78	1) M 2) F	Chronic smoker Chronic smoker	A 5 × 4 cm violaceous tender expanding mass is seen on the right upper back. Had left lung mass was 4 months prior to diagnosis. A 2 × 2 cm ulcerated, elevated, wart-like painful nodule on the right palm has been noted for two weeks ago.	CT chest-showed a left lung mass measuring 7.5 × 5.7 A chest X-ray revealed an ill-defined opacity in the right perihilar region. CT- showed a large 5.4 cm mass-like density	SCC (keratinizing type) MDSCC (keratinizing type)	p63 and CK 5/6 +ve. TTF1, CK7, and CK20 -ve P40 +ve
Xie LL et al. [18]	74	M	NA	Lung cancer developed a nodule on the puncture site of peripherally inserted central catheter.	1 cm × 1 cm in size and soft in texture, whose color was similar to that of the skin; the surface was smooth and integral without tenderness, bleeding, or exudates.	Metastatic ACC	PCK, TTF-1 +ve, CK5/6, P63, CD30, LCA, and CgA are negative.
Koyuncuer A et al. [19] Case series of 3	1) 60 2) 69 3) 55	M M M		Cases of men who presented with scalp metastasis, solitary periumbilical, and sacral as the first manifestation of an underlying lung malignancy.	NAD	Metastatic ACC. The other two were Undifferentiated NSCLC	All cases were Pan CK, CK7 +ve CK 20, TTF1 -ve
Abdeen Y. et al. [20]	72	M	Non-Smoker	A large, ulcerative, well-circumscribed lesion, measuring 2.5 cm in diameter on the right parietal scalp, progressive dyspnea over three weeks. Chest examined, showed fine bilateral diffuse crackles.	On CT, the chest shows diffuse fibrotic changes bilaterally, with multiple densities of different diameters.	ACC	CK7, CK20 +ve
Hussain J. et al. [21]	59	M	Chronic smoker	Rib pain for 1 month, a 1.5 cm right cervical lymph node, and a 0.9×0.9 cm scalp nodule.	A chest X-ray revealed a 5.8×5.0 cm left infrahilar mass. CT—extensive metastatic mediastinal and right hilar adenopathy as well as hepatic, right adrenal, and brain lesions.	Scalp and lymph node FNA shows NSCLC metastasis.	NA
Babacan NA, et al. [22]	55	M	Chronic smoker	Multiple cutaneous, painless, fragile, dark purple-colored lesions on the body measuring 1-3 cm, with a tendency to bleed.	CT chest- revealed a left hilar mass with 5.5 × 3.5 × 6 cm size with liver and bone metastasis.	SCC	P6+ve, TTF1 -ve
Grath RB. et al. [23]	61	M	Chronic smoker	Right pleuritic chest pain, 15 kg weight loss, shortness of breath, and two painful scalp lesions, both suggestive of primary lung cancer.	CT-osseous metastases and right lung apical scarring. and marrow infiltration by poorly differentiated tumor.	PDSCC	NA
Salemis NS et al. [24]	74	M	Heavy smoker	painless, movable, non-ulcerated nodule in the right temporal region measuring approximately 2 cm in diameter.	X-ray chest: a large mass in the upper left lung lobe. CT—a large mass in left upper lobe with extensive mediastinal lymphadenopathy CT head: three metastatic brain lesions.	Small-cell-carcinoma	TTF1, CK 8.18 +ve CD56, synaptophysin focally +ve
Pathak S. et al. [25]	65	M	Chronic smoker	Multiple cutaneous nodules all over the body for two months, varying in size, ranging from 2 to 6 cm, and were firm, non-tender, and mobile.	Chest X-ray (CXR) and computed tomography (CT) showed neoplastic right lung and bronchial mass with secondary cavitation.	MDSCC	NA
Wen-Hao L. et al. [26]	51	M	Smoker	Known case of right lower Lung ACC with new-onset zosteriform skin metastasis over T3-T5 dermatomes	NA	ACC	CK 7, TTF1, P53 +ve CK20, CK14, CEA-ve
Index Case	65	M	Chronic Smoker	Multiple skin nodules over abdomen for 6 months with no other specific complaints apart from having a mild backache	Ill-defined heterogeneous enhancing hypo-dense lesion with speculated margins in the left lower lobe measuring 2.6x18 mm. Pan-acinar and centri-acinar emphysematous changes diffusely involving various segments of both lungs, with the largest bulla measuring 12 cm in the right middle lobe. A few enlarged pre-tracheal, sub-carinal, and hilar lymph nodes were noted. Schmorl nodes were noted in D9 & D11 vertebrae.	ACC	CK7 +ve CK20-ve TTF1-ve Synapto-ve

Including the index case, we have a total of 19 case reports. Kocher et al. [27] evaluated 2,293 non-small cell LC (NSCLC) cases in the cohort study and found that the highest incidence was in men (70.3%) with common presenting complaints of cough (54.7%) and dyspnea (45.3%). Brownstein et al. [1] examined 724 patients and found a common occurrence of skin metastases in men (24%) due to LC, followed by colorectal carcinoma (19%), melanoma (13%), and oral SCC (12%), whereas the most common in women was breast cancer (69%), followed by colorectal carcinoma (9%), melanoma (5%), and ovarian carcinoma (4%).

Usually, cutaneous metastatic lesions from LC may be mobile or fixed, hard or flexible, single or multiple, sometimes presenting as papular, plaque-like, ulcerated, vascular, or zosteriform erysipelas-type lesions and scarring alopecia on the scalp [28]. Lesions may sometimes differ from flesh-colored to red, pink, or bluish-black, and the sizes range from 2 mm to 6 cm in diameter.

Cutaneous metastases from the lung are frequently moderately to poorly differentiated, most commonly from the adenocarcinoma subtype, followed by other subtypes: squamous cell carcinoma (SCC), small-cell carcinoma (S-CC), and large-cell carcinoma (LCC) [29]. Microscopically, metastasis from LAC displays well-differentiated glandular structures or intracytoplasmic mucinous material sometimes. SCC displays nests and sheets of squamous cells with eosinophilic cytoplasm and keratin pearls. LCCs present as undifferentiated tumors with large cells [1]. S-CC cells show features of anaplasia and display a hyperchromatic nucleus with scant cytoplasm. Clinical information, IHC, and electron microscopy (EM) features of dense core neurosecretory granules may help to distinguish S-CC from other histomorphologically similar cancers. Other primaries that need to be ruled out are from the GI tract, ovaries, kidneys, and breasts before confirmation of the diagnosis. Diagnosis is usually based upon complete clinical information, histology, IHC, and EM if required. If there is no known primary site, one must decipher whether a lesion is primary or secondary. The histological subtype could help narrow down the differentials. Multiple screening methods can be employed to determine the diagnosis, which includes complete blood count (CBC), comprehensive metabolic panel (CMP), chest X-ray, mammogram, ultrasound (US), computed tomography (CT), and magnetic resonance imaging (MRI). Solitary cutaneous metastasis is usually treated by surgery alone or in combination with chemotherapy and/or radiotherapy.

Shoenlaub et al. [30] conducted a retrospective analysis and found that LCC metastasized more frequently than was assumed for LC when compared to squamous cell carcinoma (SCC). Tamura et al. [31] predicted that out of 729 (47.3%) NSCLC cases with metastasis, the AC subtype accounted for 543 (74.5%), followed by SCC in 162 (22.2%) and LCC in 19 (2.6%).

IHC markers prove to be a useful tool for the identification of the occult primary and confirmation of cancer subtype. Markers utilized in identifying lung primary are TTF-1, CK7/20, p40, and p63. Cancers of the lung, breast, ovary, thyroid, salivary gland, and pancreas are usually positive for CK7, whereas cancers from the colon, stomach, and Merkel cells are CK20 positive. IHC markers, CK7+ and CK20-, are sensitive for primary LAC but not specific, while the anti-TTF1 marker is both sensitive and specific; hence, it is considered more reliable. However, it shows positivity in AC arising from various other organs, like primaries from the colon, endometrium, endocervix, and ovary, which must be ruled out before arriving at the diagnosis. Approximately 20% of primary LAC is TTF-1 negative. Napsin A is slightly more sensitive in detecting primary LAC. It is also proven in other studies that Napsin A staining of the primary lung is lost once it metastasizes to a distant site, which could be a finding in our case [32]. It could not be confirmed, as the patient died three months after the diagnosis was obtained.

Among 18 case reports on cutaneous metastasis from lung cancer, which included two case series with 2 and 3 cases each, 11 reported the AC subtype, five reported SCC, one reported s-CC, and four were classified as non-small cell lung cancer (NSCLC) (not subtyped). IHC was unavailable in three of the cases. TTF1 was negative in six of the total cases. Out of 11 AC, three cases were negative for TTF1, IHC was unavailable in one, and the remaining seven were positive (Table 1). Hence, our case would be the fourth reported case of TTF1-negative AC metastasis to the skin.

TTF1 is a useful marker to distinguish primary from metastatic AC. It is negative in most metastatic cases and is also a prognostic marker [33]. Cordoba JF et al. [34] study illustrated that there is shorter and worse OS for TTF1-negative NSCLC patients, regardless of treatment intention, and a lower frequency of EGFR mutations. In the index case, the patient succumbed to his illness within three months of diagnosis. In Zhang Y et al.’s [35] study, TTF1-negative LAC tumors correlated with solid and invasive mucinous-predominant subtypes (both p < 0.001), whereas TTF1+ depicted AC in situ, minimally invasive, and lepidic-predominant subtypes. The TTF1-negative defines the LAC group with recurrences and unfavorable outcomes.

Mollet TW et al. [9] performed a Medline search and found the lung as the most common primary site involved (24%) among cancers with cutaneous metastasis in men, followed by other carcinomas from the colon (19%), melanoma in the skin (13%), and the oral cavity (12%), and it was the fourth most common site (4%) in women after breast (69%), followed by other cancers from the large intestine (9%), skin (5%), and ovary (4%). TTF1 and CK7/20 markers were helpful in the diagnosis.

Cutaneous metastases from lung primaries are usually incurable and indicate poor prognosis in the form of difficult tumor resection, small-cell primary tumors, and several cutaneous or distant metastases. Mean survival is usually about 5-6 months after the diagnosis of cutaneous metastasis [9]. If the cutaneous lesion is solitary, treatment usually includes surgery alone or combined therapy and/or radiation, but chemotherapy is the primary option if lesions are more disseminated; however, it may elicit an inadequate response [22]. Whether radiation is combined with chemotherapy and/or surgery, despite the combined modality of treatment, patients with LC with cutaneous metastases have poor outcomes.

## Conclusions

Cutaneous metastasis is an unusual manifestation of LC, usually associated with advanced-stage disease, and sometimes it can be very nonspecific. It may not display any characteristic pattern on presentation; therefore, it is essential to cautiously investigate such patients with skin lesions who may turn out to have cutaneous metastasis from lung cancer, and it is essential to inquire about smoking history. Excisional lung biopsy is used both for confirmation of the diagnosis and to decide on a curative or palliative treatment of the primary tumor, given that the location and tumor characteristics allow for a safe procedure. Skin metastases at the time of presentation indicate a poor prognosis and mandate early investigation and evaluation.
